# Surface display of Lys0859, a *Streptococcus suis* prophage lysin, on *Bacillus subtilis* spores and its antibacterial activity against *Streptococcus suis*

**DOI:** 10.3389/fmicb.2025.1519935

**Published:** 2025-03-24

**Authors:** Linkang Wang, Xiaochao Duan, Mengyuan Zhu, Haiyan Wang, Xinxin Li, Dayue Hu, Xiangmin Li, Ping Qian

**Affiliations:** ^1^National Key Laboratory of Agricultural Microbiology, Hubei Hongshan Laboratory, Huazhong Agricultural University, Wuhan, China; ^2^College of Veterinary Medicine, Huazhong Agricultural University, Wuhan, China; ^3^Key Laboratory of Preventive Veterinary Medicine in Hubei Province, The Cooperative Innovation Center for Sustainable Pig Production, Wuhan, China; ^4^Hubei Jiangxia Laboratory, Wuhan, China

**Keywords:** *Streptococcus suis* prophage lysin, *Bacillus subtilis*, CotG, spore surface display, *Streptococcus suis* infection

## Abstract

**Introduction:**

*Streptococcus suis*, an important zoonotic and opportunistic pathogen in pigs, brings huge economic losses to the pig-raising industry and infects humans with diseases. Phage lysin is regarded as a promising substitute for antibiotics due to its ability to quickly and efficiently kill bacteria without easily developing resistance. However, their clinical applications have been hindered by inherent instability under environmental stressors.

**Methods:**

We constructed *B. subtilis* spores displaying bacteriophage lysin Lys0859 using spore coat protein CotG as an anchoring motif. Environmental tolerance was evaluated through thermal (37–95°C), pH (1.0–8.0), and enzymatic challenges, while antibacterial efficacy against *S. suis* was assessed using agar diffusion assays and murine infection models with systemic bacterial load quantification.

**Results:**

The spore-display system enhanced environmental resistance of Lys0859 while preserving its bactericidal efficacy. In vitro assays demonstrated 1 × 10^6^ CFU rBS^CotG-0859^ spores exhibited equivalent bactericidal activity to 39.11 μg free Lys0859 against *S. suis*. *In vivo*, spore treatment reduced *S. suis* SC19 colonization by 0.47–1.96 log units (*p* < 0.05) across all tissues compared with PBS controls.

**Discussion:**

This study achieved functional display of prophage lysin Lys0859 on *B. subtilis* spores through CotG anchoring, demonstrating potent *in vitro* anti-streptococcal activity. Crucially, this strategy streamlined bioproduction by eliminating purification demands and lowering costs, lays the foundation for the clinical application of prophage lysin.

## 1 Introduction

*Streptococcus suis* (*S. suis*) is an important zoonotic pathogen responsible for infecting both humans and a wide range of animal species. *S. suis* can spread and infect through contact with infected animals or contaminated animal products, causing serious threats to the pig industry (Wang et al., 2022) and public health security worldwide (Feng et al., 2010). In China, there were two large-scale human infections of *S. suis* in 1998 and 2005, resulting in 53 deaths, which attracted great social attention (Normile, 2005; Tang et al., 2006). So far, 29 serotypes of *S. suis* have been identified based on capsular polysaccharide, among which serotype 2 is the serotype with the highest clinical isolation rate and is considered to be one of the most virulent serotypes (Goyette-Desjardins et al., 2014; Liu et al., 2019; Okura et al., 2016). *S. suis* mainly inhabits the upper respiratory tract of pigs, especially the tonsils and nasal cavities, as well as the digestive and genital tracts (Goyette-Desjardins et al., 2014). After humans or pigs are infected with the virulent strains of *S. suis*, they often exhibit meningitis, septicemia, arthritis, endocarditis, pneumonia, and other diseases (Gottschalk and Segura, 2000; Segura et al., 2017). Like most other bacterial infections, antibiotic therapies remain one of the most effective methods for *S. suis* infection. However, an increasing number of studies have reported that the prolonged use or misuse of antibiotics can lead to environmental pollution, antibiotic residues, and the production of multidrug-resistant bacteria (Brown and Wright, 2016; Zhang et al., 2018; Hall et al., 2020). Therefore, there is an urgent need to find a novel antibacterial drugs or antibiotic alternatives to combat the infection of *S. suis*.

Phage lysin is a cell wall hydrolase encoded by bacteriophage, which can rapidly destroy the cell wall structure by degrading peptidoglycans in the cell wall upon contact with the host cells, and release newly assembled bacteriophages (Young et al., 2000). Compared with traditional antibiotics, bacterial resistance to lysin is difficult to develop, and lysin can specifically lyse target pathogens without disturbing commensal flora (Wang Z. et al., 2019). Therefore, lysin as a promising antibacterial agent has been studied for various biomedical applications, and its effectiveness has been conformed in multiple animal model of infected with resistant bacteria (Gilmer et al., 2013; Eichenseher et al., 2022). However, lysin is sensitive to multiple environmental factors, such as temperature, pH, and proteases, which affect their bioactivity and thus limit the bactericidal effect in clinical practice. Therefore, there is an emergent search for a method to enhance the resistance of lysin to cope with environmental stresses. *B. subtilis* spore surface display is considered one of the most effective ways to deliver heterologous proteins with high biological activity and stability (Iwanicki et al., 2014). The United States Food and Drug Administration (FDA) has classified *B. subtilis* as a Generally Recognized as Safe (GRAS) strain. It can be used as a food additive in food preparations for both humans and animals, and is also applicable in the treatment of gastrointestinal diseases (Cutting et al., 2009). *B. subtilis* spores offer several advantages in anchoring heterologous proteins: (1) *B. subtilis* spores exhibit greater adaptability to extreme environments, including nutrient deprivation, drastic temperature and pH fluctuations, ultraviolet irradiation, and toxic substances (Ullah et al., 2024). (2) Heterologous proteins anchored on the spore surface do not need to cross the cell membrane. This not only avoids protein misfolding but also ensures the structure and biological activity of the exogenous proteins (Guo et al., 2018). (3) Enzymes displayed on the spore surface generally possess good reusability and stability (Guo et al., 2018). For instance, the p75 protein was displayed on the surface of *B. subtilis* spores using spore coat protein CotG as an anchoring motif, and the peptidoglycan hydrolase activity, stability, and the antibacterial activity of the spore-displayed p75 protein were significantly enhanced (Kang et al., 2020). However, we have hardly found any reports on the display of prophage lysin on the spore surface of *B. subtilis*.

In this study, the prophage lysin Lys0859 from *S. suis* SS0859 was successfully exhibited on the spore surface by employing the outer coat protein G (CotG) of *B. subtilis* spores as the anchor protein. Then, we evaluated the *in vitro* resistance of Lys0859 displayed on the spore surface of recombinant bacteria against environmental assaults, as well as antimicrobial spectrum of the recombinant bacteria *in vitro*. Furthermore, we explored the protective effect of recombinant bacteria on *S. suis* SC19 infection by murine model of systemic *S. suis* infection.

## 2 Materials and methods

### 2.1 Bacterial strains and growth conditions

The bacterial strains used in this study are listed in Supplementary Table S1, and plasmids and primers are listed in Supplementary Table S2. The *streptococci* strains were cultured in tryptic soy broth (TSB) (BD Biosciences, MD, United States) supplemented with 5% (v/v) fetal bovine serum (Solarbio, Beijing, China) or TSB plates containing 1.5% (w/v) agar and 5% (v/v) fetal bovine serum at 37°C. The other strains were cultured in Luria–Bertani (LB) broth or LB plates containing 1.5% (w/v) agar at 37°C*. B. subtilis* sporulation was cultured in Difco sporulation medium (DSM), as previously described (Mascarenhas et al., 2002).

### 2.2 Construction of CotG-0859 fusion protein expression plasmid

To display phage lysin 0859 (Lys0859), which was previously discovered in our laboratory (Li et al., 2023), on the surface of *B. subtilis* 168 spores, we constructed a CotG-0859 fusion. The CotG gene was amplified with primers CotG-F/CotG-R from the *B. subtilis* 168 chromosomes. The Lys0859 gene from *S. suis* 0859 was amplified using primers 0859-F/0859-R by PCR. Then, the CotG and 0859 genes were constructed into a CotG-0859 fusion by overlap-extension PCR, digested with *BamH* I and *EcoR* I (Takara, China), and ligated into pDG364 vector to obtain the recombinant plasmids pDG364-CotG-0859. Meanwhile, pCold-CotG-0859 (CotG-F/CotG-R, 0859-F/0859-R1, BamH I/Hind III) and pCold-0859 (0859-F1/0859-R1, BamH I/Hind III) were constructed using the same methods. Recombinant plasmids were transformed into *E. coli* DH5α and the positive colonies were identified by colony-PCR.

### 2.3 Construction of recombinant *Bacillus subtilis* 168

The obtained pDG364-CotG-0859 recombinant plasmid was linearized by *Kpn* I (Takara, China) enzyme digestion, and transformed into the amylase E (*AmyE*) gene of the competent *B. subtilis* genome by the natural transformation method (Spizizen, 1958). Then, the clones generated after the integration of the target gene at the *AmyE* locus of *B. subtilis* were selected on LB agar plates supplemented with 5 μg/mL chloramphenicol. Used LB agar plates containing 1% starch to screen positive strains with amylase gene deletion. Extracted recombinant bacterial genomic DNA and used AmyE-F/AmyE-R, G8-F/G8-R, G8-F/AmyE-R, and AmyE-F/G8-R for PCR identification, respectively.

### 2.4 Spore preparation

The sporulation of wild-type *B. subtilis* 168 (WT) and recombinant *B. subtilis* were induced at 37°C for 48 h using Difco Sporulation Medium (DSM). Spore purification was performed as previously described, with some modifications. The spores were collected by centrifugation 8,000 g for 10 min at 4°C, and washed with 0.5 M NaCl. Resuspend the spores in Tris–HCl (50 mmol/L, pH 7.2) containing a final concentration of 50 μg/mL of lysozyme and incubate at 37°C in a water bath for 1 h. Then, the spores were harvested by centrifugation and washed with 1M NaCl, 1M KCl, and distilled water (three times), respectively. After incubation at 65°C for 1 h, pure spores were harvested and resuspended in sterile PBS, and stored at −40°C.

### 2.5 Expression of fusion proteins in *Bacillus subtilis* and SDS-PAGE and western blot analysis

The purified spores were incubated with SDS-DTT extraction buffer at 70°C for 30 min to extract spore coat proteins. To confirm that the surface display of Lys0859 on the spore coat, the extracted spore coat proteins was subjected to SDS-PAGE. Subsequently, one portion was stained with Coomassie Brilliant Blue, while the other portion was transferred to polyvinylidene fluoride (PVDF) membrane. The membrane was incubated with PBST containing 5% skim milk for 2 h. After three washes with PBST, incubates with Lys0859 antiserum (1:5,000 in PBST) for 2 h. Then, the membrane was incubated with HRP-conjugated Goat anti-Mouse IgG second antibody (1:5,000 in PBST) for 1 h at room temperature and visualized by ECL detection.

### 2.6 Immunofluorescence microscopy

To detect Lys0859 on the surface of spores, 1 mL of purified spore solution was harvested after 48 h of *B. subtilis* induction and fixed on a glass slide, and blocked with 5% BSA for 2 h. Anti-Lys0859 antibody (1:400 in PBST) was added and incubated for 2 h at room temperature. Then, FITC labeled goat anti-mouse IgG antibody (1:400 in PBST) was added and the spores were observed with a fluorescence microscope (Olympus, Japan).

### 2.7 The growth curves and sporulation rate of recombinant *Bacillus subtilis*

To investigate whether the insertion of exogenous proteins affects the activity of the strain, we determined the growth curves and sporulation rate of *B. subtilis* 168 and rBS^CotG-0859^. *B. subtilis* 168 and rBS^CotG-0859^ were inoculated into LB medium or DSM sporulation medium (final concentration 1 × 10^4^ CFU/mL) respectively, and incubated at 37°C with shaking (180 rpm). Samples were collected every 2 h to prepare 10-fold serial dilutions and spread onto LB agar plates and cultured at 37°C for 12 h before bacterial counting. For spore counting, the spore suspensions were treated at 85°C for 5 min, and then 10-fold serial dilutions were prepared to place on LB agar plates and cultured at 37°C for 12 h before counting. The sporulation rate was calculated with the following equation: Sporulation rate = number of spores/ number of vegetative cells.

### 2.8 *In vitro* resistance assay of recombinant spores

The rBS^CotG-0859^ spores (1 × 10^7^ CFU) were separately resuspended into 1 mL of sterile PBS with different pH values (1, 2, 3, 4, 5, 6, 7, or 8), or simulated gastric fluid (SGF, HCl, pH 1.2) containing 10 g/L of pepsin in 0.85% NaCl solution, or simulated intestinal fluid (SIF, NaOH, pH 6.8) containing 10 g/L of trypsin in 0.05 M KH_2_PO_4_ solution, and incubated at 37°C. At predetermined time points, the samples were centrifuged at 6,000 g for 5 min at 4°C, and bacterial cells were washed twice with sterile PBS and resuspended in 100 μL of PBS. Next, bacteriostatic activity of rBS^CotG-0859^ spores (1 × 10^7^ CFU) against *S. suis* SC19 was tested by the agar-well diffusion assays. The plates were incubated at 37°C for 12 h, and the diameter of inhibition zone was measured with vernier caliper.

One milliliter of rBS^CotG-0859^ spores (1 × 10^7^ CFU) were separately placed in water baths at different temperatures (37°C, 45°C, 55°C, 65°C, 75°C, 85°C, or 95°C) for 30 min. Bacterial cells were then collected by centrifugation (6,000 g, 5 min, 4°C), washed twice and resuspended in 100 μL sterile PBS. The bacteriostatic activity was measured as described above.

### 2.9 Genetic stability of recombinant spores

To assess the genetic stability of recombinant bacteria, the rBS^CotG-0859^ were serially passaged on LB agar plates up to 10 times. A single bacterial colony was picked and inoculated into 1 mL of LB medium (contains 5 μg/mL chloramphenicol) and incubated overnight at 37°C with shaking (180 rpm). PCR identification of rBS^CotG-0859^ clone was performed using 168-F/168-R and G8-F/G8-R primers, respectively. The rBS^CotG-0859^ (the 1st to 10th generation colonies) were induced to sporulate in DSM for 48 h at 37°C and purified them. The antibacterial activity of rBS^CotG-0859^ (the 1st to 10th generation) against *S. suis* SC19 by agar diffusion assay. All plates were cultured at 37°C for 12 h before observing the inhibition zone, and the diameter of the inhibition zone was measured using a vernier caliper. The presence of a clear zone indicated antagonistic activity.

### 2.10 *In vitro* antibacterial activity of recombinant spores

The *in vitro* antagonistic activity of rBS^CotG-0859^ spores was tested using the agar well-diffusion method against common pathogens. The bacterial lawns were prepared by mixing 10 mL of TSB medium containing 5% fetal bovine serum (FBS) with bacterial culture suspension (1 × 10^6^ CFU/mL) and then poured onto a sterile plate covered with LB agar and Oxford cups, wells of 8 mm diameter were made on TSB agar after removing the cups. The rBS^CotG-0859^ spores and different dosages of Lys0859 in a total volume of 100 μL were then added separately into the well on the agar plate. After incubating at 37°C for 12 h, and measuring the diameter of inhibition zone. The diameter of the inhibition zone (mm) was the average of three independent experiments and presented in the form of a bar graph. The five known doses of Lys0859 and their corresponding inhibition zone sizes were displayed on the x-y scatter plot, respectively. Thereafter, the best-fit linear regression equation (LRE) was plotted based on the linear trend line. After inhibition zone diameter (rBS^CotG-0859^) were fitted in the LRE, the equivalent dose of lysis enzyme activity of the recombinant bacteria was ultimately obtained.

### 2.11 *In vivo* study of bactericidal activity of recombinant spores

A model of *S. suis* SC19 infection mouse was established using 4-week-old specific pathogen-free (SPF) female ICR mice purchased from the Experimental Animal Center of Huazhong Agricultural University, Wuhan, China. Mice were randomly divided into 4 groups (*n* = 6): (i) control; (ii) SC19 + PBS group; (iii) SC19 + BS168; (iiii) SC19 + rBS^CotG-0859^. On the first experimental day, all mice were injected intraperitoneally with 100 μL of SC19 (6 × 10^7^ CFU/mouse) in the SC19 + PBS group, SC19 + BS168 group, and SC19 + rBS^CotG-0859^ group. On days 1, 2, 3 and 4 following SC19 infection, mice in the SC19 + rBS^CotG-0859^ group and SC19 + BS168 group separately received 200 μL (2 × 10^7^ CFU/mouse) of rBS^CotG-0859^ spores and BS168 spores via gavage administration. In contrast, the mice in the control group and the SC19 + PBS group were administered the same volume of sterile PBS. Fresh fecal samples were collected daily following SC19 infection and directly resuspended in sterile PBS. The number of rBS^CotG-0859^ in mice feces from the SC19 + rBS^CotG-0859^ group was then determined by spreading a series of 10-fold dilutions on LB agar plates containing 10 μg/mL chloramphenicol. The general health of all mice was monitored on a regular daily throughout the experiment. On the 7th day after SC19 infection, all mice were sacrificed by cervical vertebra dislocation, the blood and the major organs (heart, liver, spleen, lung, kidney, and brain) were collected and fixed with 4% paraformaldehyde. Then, the remaining organ tissues were weighed and homogenized. The number of SC19 in the heart, liver, spleen, lungs, kidneys, brain, and blood were determined by spreading a series of 10-fold dilutions on TSA plates.

To further study the prophylactic efficacy of rBS^CotG-0859^ spores against SC19 infection, another independent experiment was conducted using four-week-old female ICR mice (SPF). Twenty-four mice were randomly divided into four groups (n = 6): control group, PBS + SC19 group, BS168 + SC19 group and rBS^CotG-0859^ + SC19 group. On days 1 to 7, each mouse in the rBS^CotG-0859^ + SC19 group received 200 μL (2 × 10^7^ CFU/mouse) of rBS^CotG-0859^ spores via gavage once a day. At the same time, mice in the BS168 + SC19 group received an equal amount of BS168 spores, while intragastric gavages with 200 μL of sterile PBS to mice of the control group and PBS + SC19 group. On day 7, all mice in the PBS + SC19 group, BS168 + SC19 group, and rBS^CotG-0859^ + SC19 group were injected intraperitoneally with 200 μL of SC19 (6 × 10^7^ CFU/mouse). Fresh fecal samples were collected daily following SC19 infection and directly resuspended in sterile PBS. The number of rBS^CotG-0859^ in feces of mice from the rBS^CotG-0859^ + SC19 group was determined referring to the method described in the rBS^CotG-0859^ therapeutic trial. Throughout the study, the health of the mice was monitored on a regular daily. On day 12, all mice were sacrificed by cervical vertebra dislocation, the blood and the major organs (heart, liver, spleen, lung, kidney, and brain) were collected and fixed with 4% paraformaldehyde. Then, the remaining organ tissues were weighed and homogenized, and the number of SC19 in the heart, liver, spleen, lungs, kidneys, brain, and blood was measured using TSA plates.

All animal experiments were performed with the approval of the Scientific Ethic Committee of Huazhong Agricultural University (no. HZAUMO-2024-0075).

### 2.12 Statistical analysis

Statistical analysis was performed using GraphPad Prism 8.3.0 (GraphPad Software, San Diego, CA, United States). Data were expressed as mean ± standard deviation (SD), and analysis comparisons were carried out using one-way analysis of variance (ANOVA) followed by Tukey’s multiple-comparison test (*p < 0.05, **p < 0.01, and ***p < 0.001).

## 3 Results

### 3.1 Construction of recombinant plasmids and bacterial strains

**Figure 1:**
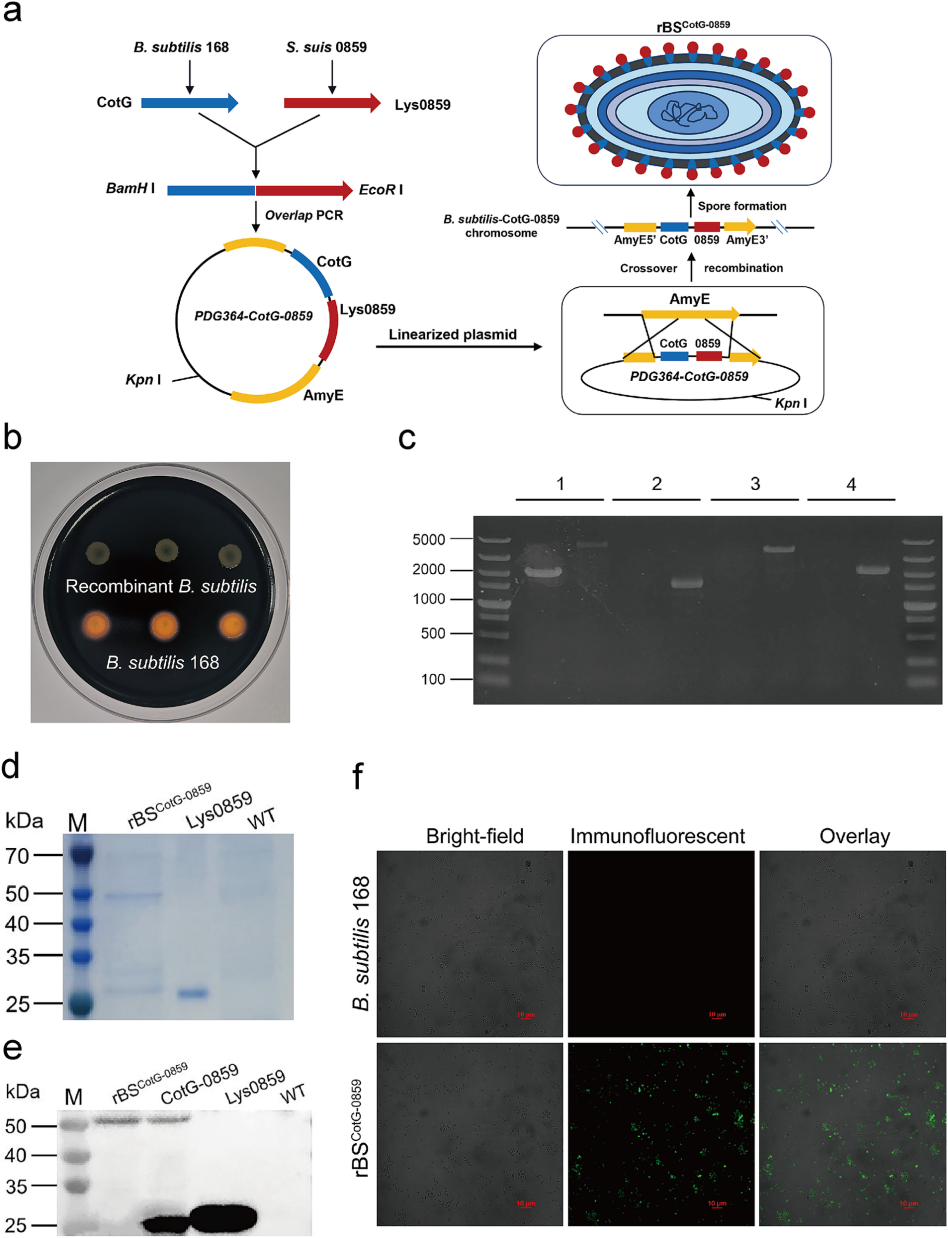
Construction of recombinant *B. subtilis* with surface display of phage lysin 0859. **(a)** The construction process of recombinant *B. subtilis* with surface display of Lys0859 on the surface (rBS^CotG-0859^). **(b)** Starch hydrolysis test. *B. subtilis* 168 and rBS^CotG-0859^ were cultured on a LB-agar medium containing 1% starch and stained with iodine. **(c)** PCR analysis of *B. subtilis* using different primer pairs: (1) AmyE-F and AmyE-R; (2) G8-F and G8-R; (3) G8-F and AmyE-R; (4) AmyE-F and G8-R. In the results displayed for each primer pair, the left lane represents *B. subtilis* 168 and the right lane represents rBS^CotG-0859^. **(d)** SDS-PAGE and **(e)** western blot analysis of proteins extracted from spores of the *B. subtilis* wild-type (WT) strain and rBS^CotG-0859^; and Lys0859 or CotG-0859 proteins obtained from prokaryotic expression were used as a control; **(f)** Immunofluorescence of CotG-0859 on the spore surface at 48 h after induction of wild-type (WT) and rBS^CotG-0859^. The scale bar represents 10 μm.

To validate whether Lys0859 displayed on the spore surface enhances antibacterial activity or tolerance, we constructed a recombinant *B. subtilis* strain (rBS^CotG-0859^) expressing Lys0859 on its spore surface. Briefly, the exogenous fusion gene CotG-0859 was inserted into the amylase gene of *B. subtilis* through homologous recombination, resulting in the inactivation of the amylase gene (Figure 1a). Therefore, recombinant bacteria (rBS^CotG-0859^) could not hydrolyze starch compared to the wild-type (Figure 1b). To further verify whether CotG-0859 was successfully inserted into the *AmyE* gene, PCR were performed using 4 sets primers. The results of agarose-gel electrophoresis showed that the bands amplified from the genome of rBS^CotG-0859^ were 4,448 bp, 1,544 bp, 3,831 bp, and 2,161 bp from left to right, which was consistent with the expected size (Figure 1c). We used Coomassie blue staining to identify the capsid proteins extracted from the rBS^CotG-0859^ spores. A specific band with molecular weight of 51 kDa appeared in the extracts of rBS^CotG-0859^ spores, but extract from wild strains (*B. subtilis* 168) did not (Figure 1d). In addition, western blot results showed that a positive hybridization band with 51 kDa was detected in the capsid proteins extracted from the rBS^CotG-0859^ spores. Conversely, it was not detected in the extracts of wild strains (*B. subtilis* 168) (Figure 1e). The surface expression of the fusion protein was also confirmed by immunofluorescence (IF), with strong fluorescence was observed on rBS^CotG-0859^ spores compared to wild-type spores (Figure 1f).

### 3.2 The growth and sporulation rate of rBS^CotG-0859^ and its *in vitro* resistance assay

**Figure 2:**
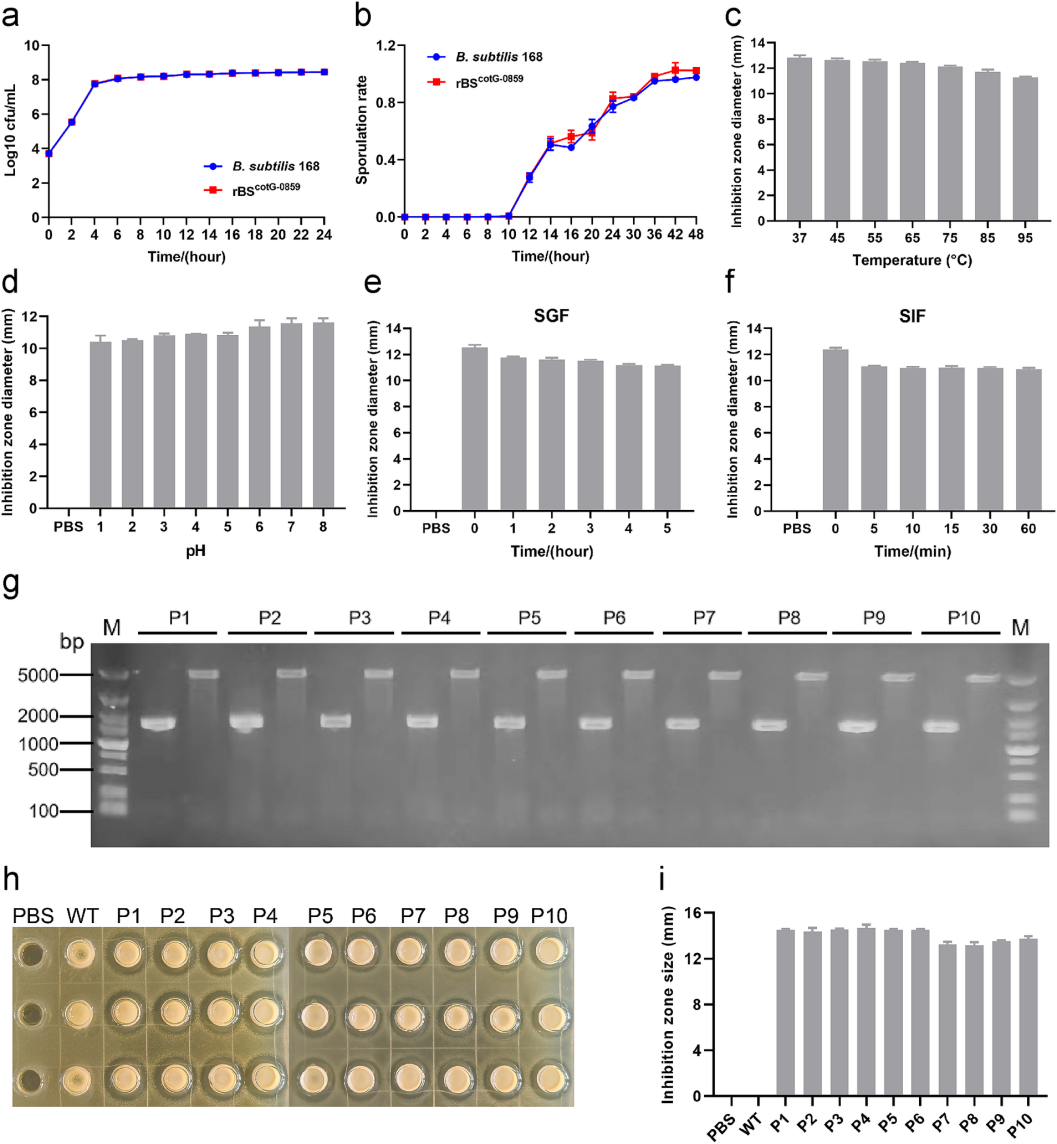
The growth and sporulation rate of rBS^CotG-0859^ and resistance assay *in vitro*. **(a)** Growth curves of *B. subtilis* 168 and rBS^CotG-0859^. **(b)** The sporulation curves of *B. subtilis* 168 and rBS^CotG-0859^. Effects of **(c)** different temperatures, and **(d)** different pH on the antibacterial activity of rBS^CotG-0859^ spores. The rBS^CotG-0859^ spore (1 × 10^7^ CFU) was exposed to **(e)** SGF supplemented with gastric protease (pH 1.2) and **(f)** SIF containing trypsin (pH 6.8). At predetermined time points, bacteriostatic activity of rBS^CotG-0859^ spores (1 × 10^7^ CFU) against *S. suis* SC19 was tested by the agar-well diffusion assays. The diameter of inhibition zone was measured after incubation for 12 h at 37°C. **(g)** PCR analysis of the 1st to 10th generation rBS^CotG-0859^ using different primer pairs: G8-F and G8-R; 168-F and 168-R. **(h)** The antibacterial activity of rBS^CotG-0859^ (the 1st to 10th generation) against *S. suis* SC19 after passage. **(i)** The size of each inhibition zone shown in figure H was measured using a vernier caliper, and presented as a bar chart.

The growth curve and sporulation rate of *B. subtilis* 168 and rBS^CotG-0859^ were determined through a standard plate-counting method. As shown in Figure 2a, the growth curve of the rBS^CotG-0859^ was always consistent with the *B. subtilis* 168, indicating that the insertion of exogenous DNA did not affect the growth of the recombinant strains. In addition, the high-level expression of heterologous proteins did not have a significant effect on the sporulation rate of rBS^CotG-0859^ compared with the *B. subtilis* 168 (Figure 2b).

Moreover, we evaluated the effects of temperature, pH, SGF, and SIF on the antibacterial activity of rBS^CotG-0859^ spores. As shown in Figure 2c, the antibacterial activity of rBS^CotG-0859^ spores against *S. suis* SC19 showed a gentle downward trend with increasing temperature. Meanwhile, the antibacterial activity of rBS^CotG-0859^ spores showed a declining tendency toward when the pH decreased (Figure 2d), and still maintained excellent antibacterial activity at pH 1.0. Similar experimental results were observed in both simulated gastric fluid (pepsin 1 mg/mL, pH 1.2) (Figure 2e) and simulated intestinal fluid (trypsin 1 mg/mL, pH 6.8) (Figure 2f). Furthermore, the rBS^CotG-0859^ were serially passaged on LB agar plates up to 10 times (Supplementary Figure S1), and identified by PCR analysis. Nucleic acid electrophoresis showed that the bands amplified from the 1st to 10th generation rBS^CotG-0859^ genome were 1,544 bp and 4,854 bp (Figure 2g), which was consistent with the expected size. As shown in Figures 2h,i, the 1st to 10th generation rBS^CotG-0859^ spores exhibited good antibacterial activity against *S. suis* SC19.

### 3.3 Bactericidal activity of Lys0859 displayed by rBS^CotG-0859^ against *Streptococcus suis* SC19

**Figure 3:**
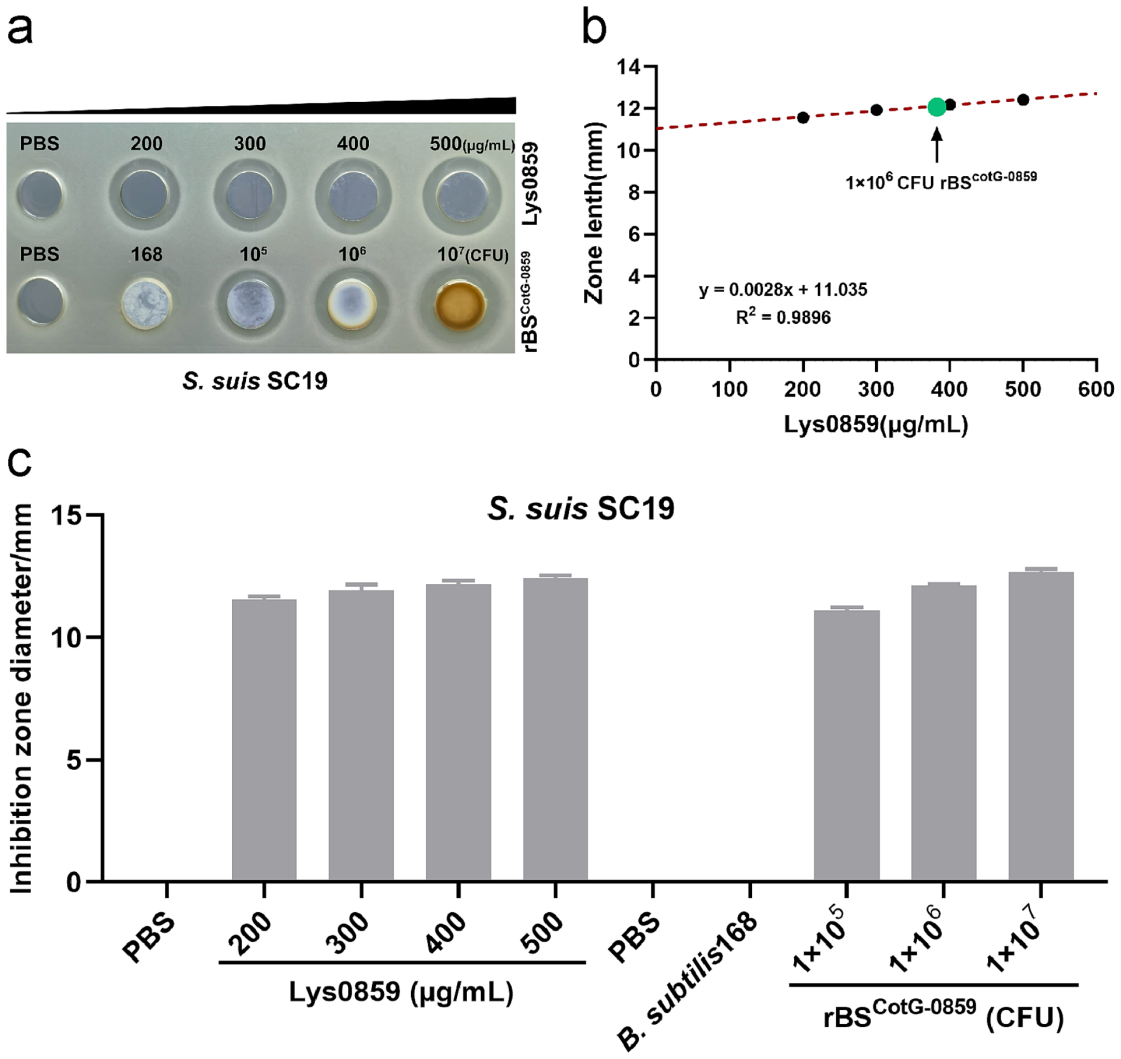
Bactericidal activity of Lys0859 displayed by rBS^CotG-0859^ against *S. suis* SC19. **(a)** The antibacterial efficacy of rBS^CotG-0859^ was determined by agar diffusion assay against *S. suis* SC19. **(b)** The regression equation was obtained based on four known dosages of Lys0859 that corresponded to their inhibition zones. Antibacterial potency equivalent to Lys0859 of rBS^CotG-0859^ spores against *S. suis* SC19 was determined. **(c)** The size of each inhibition zone shown in **(a)** was measured using a vernier caliper, and presented as a bar chart.

Following confirmation that the Lys0859 enzyme successful displayed on the surface of rBS^CotG-0859^ spores, we validated whether the rBS^CotG-0859^ spores exhibited bactericidal activity against *S. suis* SC19 through an agar-well diffusion assay. The purified rBS^CotG-0859^ spores were added into an 8-mm diameter well on agar plate confluent with *S. suis* SC19, and incubated at 37°C for 12 h. The well containing Lys0859 enzyme displayed an antibacterial zone, served as a positive control, while the spore of *B. subtilis* 168 has no antibacterial activity and used as a negative control (Figure 3a). The inhibition zone diameters (11.56, 11.92, 12.17, and 12.41 mm, respectively) of Lys0859 enzyme with different dosages (from low to high were 20, 30, 40, and 50 μg) were measured on a double-layer agar plate containing *S. suis* SC19 (Figure 3c). To better understand the relationship between dose and inhibition zone, three known dosages of Lys0859 enzyme and corresponding inhibition zones were transformed into a linear regression equation, which is y = 0.0028x + 11.035, *R*^2^ = 0.9896 (Figure 3b). After incorporating the inhibition zone diameters of rBS^CotG-0859^ spores (1 × 10^6^ CFU) into the regression equation, the antibacterial potency against *S. suis* SC19 were equivalent to 39.11 μg Lys0859 enzyme, respectively.

### 3.4 Bactericidal activity of rBS^CotG-0859^ against common pathogens

Based on the excellent antibacterial effect of Lys0859 displayed by rBS^CotG-0859^ against *S. suis* SC19, we next examined whether rBS^CotG-0859^ spores could kill clinical isolates (pathogenic bacteria) of *S. suis*. Here we still use Lys0859 as a positive control for the agar well diffusion assay, and *B. subtilis* 168 spores (1 × 10^7^ CFU) as a negative control. The results of the study demonstrated that Lys0859 exhibited a dose-dependent antibacterial effect against *streptocci*. Four dosages of Lys0859 with concentrations of 20, 30, 40, and 50 μg showed an average inhibition zone of 11.2, 12.02, 12.41, 12.66 mm (Supplementary Figure S2a), 14.15, 14.77, 14.92, 15.25 mm (Supplementary Figure S2b), 12.95, 13.3, 13.73, 13.96 mm (Supplementary Figure S2c), 12.03, 12.31, 12.74, 12.92 mm (Supplementary Figure S2d), 11.22, 11.53, 11.86, 12.17 mm (Supplementary Figure S2e), 8.98, 9.25, 9.82, 9.93 mm (Supplementary Figure S2f), and 12.65, 12.98, 13.34, 13.57 mm (Supplementary Figure S2g), respectively, on the agar plate containing *S. suis* SS3, *S. suis* 18SS35, *S. suis* SS4, *S. suis* 18SS8, *S. suis* SS19, *S. suis* 1SS3 or *S. suis* 18SS75. The rBS^CotG-0859^ spores with concentrations of 1 × 10^6^ CFU exhibited an average inhibition zone of 12.54, 14.34, 14.11 and 12.25 mm against *S. suis* SS3 (Supplementary Figure S2a), *S. suis* 18SS35 (Supplementary Figure S2b), *S. suis* SS4 (Supplementary Figure S2c) and *S. suis* 18SS8 (Supplementary Figure S2d), respectively. Additionally, the rBS^CotG-0859^ spores at a concentration of 1 × 10^5^ CFU displayed mean inhibition zone of 11.78 mm, 10.86 mm, and 13.58 mm against *S. suis* SS19 (Supplementary Figure S2e), *S. suis* 1SS3 (Supplementary Figure S2f) and *S. suis* 18SS75 (Supplementary Figure S2g), respectively. Based on the linear regression analysis (Table 1), the bactericidal activity of 1 × 10^6^ CFU rBS^CotG-0859^ spores against *S. suis* SS3, *S. suis* 18SS35, *S. suis* SS4 and *S. suis* 18SS8 were equivalent to 44.52 μg, 22.14 μg, 52.45 μg and 26.93 μg of Lys0859, respectively. The bactericidal activity of 1 × 10^5^ CFU rBS^CotG-0859^ spores against *S. suis* SS19, *S. suis* 1SS3 and *S. suis* 18SS75 were equivalent to 37.44 μg, 75.35 μg, 49.58 μg of Lys0859, respectively.

**Table 1:** The linear regression equation (LRE) of each pathogen showed on agar well diffusion assay.

Pathogenic bacteria	Enzyme used	LRE	*R* ^2^
*S. suis* SS4	Lys0859	y = 0.0035x + 12.274	0.9876
*S. suis* SS15	Lys0859	y = 0.0035x + 13.147	0.9945
*S. suis* SS19	Lys0859	y = 0.0032x + 10.582	0.9998
*S. suis* SS30	Lys0859	y = 0.0048x + 12.67	0.9332
*S. suis* 1SS3	Lys0859	y = 0.0034x + 8.298	0.9431
*S. suis* SS23	Lys0859	y = 0.0025x + 14.315	0.9642
*S. suis* 18SS8	Lys0859	y = 0.0031x + 11.415	0.9786
*S. suis* 18SS23	Lys0859	y = 0.0033x + 12.195	0.9875
*S. suis* 18SS29	Lys0859	y = 0.0034x + 13.255	0.9768
*S. suis* 18SS35	Lys0859	y = 0.0035x + 13.565	0.9339
*S. suis* 18SS75	Lys0859	y = 0.0031x + 12.043	0.9923
*S. suis* 18SS91	Lys0859	y = 0.005x + 11.805	0.9918
*S. suis* SS3	Lys0859	y = 0.0048x + 10.403	0.9302
*S. suis* 19SS9	Lys0859	y = 0.0051x + 8.799	0.9638
*S. aureus* ATCC43300	Lys0859	y = 0.004x + 9.997	0.9391
*S. agalactiae* ATCC13813	Lys0859	y = 0.0041x + 9.152	0.9965
*S. agalactiae* X2	Lys0859	y = 0.0047x + 7.97	0.9723
*S. dysgalactiae* SD002	Lys0859	y = 0.0035x + 9.205	0.9216

LRE represented the relationship between Lys0859 dose and inhibition zone shown on the agar plate. Lys0859, phage lysin 0859; R2, coefficient of correlation.

For *S. suis* SS15, *S. suis* SS23, *S. suis* SS30, *S. suis* 18SS23, *S. suis* 18SS29, *S. suis* 18SS91 and *S. suis* 19SS9, four different dosages of Lys0859 (20, 30, 40, and 50 μg) exhibited an average inhibition zone of 13.86, 14.22, 14.51, 14.94 mm (Supplementary Figure S3a), 14.75, 15.11, 15.34, 15.49 mm (Supplementary Figure S3b), 13.67, 14.17, 14.35, 15.21 mm (Supplementary Figure S3c), 12.89, 13.16, 13.46, 13.89 mm (Supplementary Figure S3d), 13.87, 14.31, 14.66, 14.87 mm (Supplementary Figure S3e), 12.85, 13.22, 13.76, 14.32 mm (Supplementary Figure S3f), and 9.72, 10.38, 10.97, 11.21 mm (Supplementary Figure S3g), respectively. The rBS^CotG-0859^ spores with concentrations of 1 × 10^7^ CFU showed an average inhibition zone of 13.57, 14.39, 12.85, 13.67, 14.43, 13.19 and 11.12 mm against *S. suis* SS15 (Supplementary Figure S3a), *S. suis* SS23 (Supplementary Figure S3b), *S. suis* SS30 (Supplementary Figure S3c), *S. suis* 18SS23 (Supplementary Figure S3d), *S. suis* 18SS29 (Supplementary Figure S3e), *S. suis* 18SS91 (Supplementary Figure S3f) and *S. suis* 19SS9 (Supplementary Figure S3g), respectively. Calculated based on the linear regression equation shown in Table 1, the bactericidal activity of 1 × 10^7^ CFU rBS^CotG-0859^ spores against *S. suis* SS15, *S. suis* SS23, *S. suis* SS30, *S. suis* 18SS23, *S. suis* 18SS29, *S. suis* 18SS91 and *S. suis* 19SS9 were equivalent to 12.09 μg, 30 μg, 3.75 μg, 44.70 μg, 34.56 μg, 27.7 μg and 45.51 μg of Lys0859, respectively.

Next, we performed another antimicrobial test on *Streptococcus agalactiae* ATCC13813, *Streptococcus agalactiae* X2 and *Streptococcus dysgalactiae* SD002. Analogous to the above experiments, different dosages of Lys0859 (20, 30, 40, and 50 μg) exhibited an average inhibition zone of 9.95, 10.42, 10.83, 11.19 mm, 8.81, 9.46, 9.91, 10.21 mm, and 9.78, 10.42, 10.65, 10.87 mm on the agar plate containing *S. agalactiae* ATCC13813 (Supplementary Figure S4a), *S. agalactiae* X2 (Supplementary Figure S4b) or *S. dysgalactiae* SD002 (Supplementary Figure S4c), respectively. The average inhibition zones of rBS^CotG-0859^ spores (1 × 10^6^ CFU) against *S. agalactiae* ATCC13813, *S. agalactiae* X2 and *S. dysgalactiae* SD002 were 10.21, 11.68, and 10.84 mm, respectively. The bactericidal activity of 1 × 10^6^ CFU rBS^CotG-0859^ spores against *S. agalactiae* ATCC13813, *S. agalactiae* X2 and *S. dysgalactiae* SD002 was equivalent to 25.85 μg, 57.65 μg and 46.71 μg of Lys0859 through linear regression analysis.

We also validated the bactericidal activity of rBS^CotG-0859^ spores against *S. aureus* ATCC43300 and found that Lys0859 with different dosages of 20, 30, 40, and 50 μg exhibited an average inhibition zone of 10.68, 11.38, 11.55, 11.95 mm (Supplementary Figure S4d), respectively. The rBS^CotG-0859^ (1 × 10^7^ CFU) spores displayed an average inhibition zone of 10.79 mm on the agar plate containing *S. aureus* ATCC43300. Based on the results of a simple linear regression analysis, it was determined that the bactericidal activity of 1 × 10^7^ CFU rBS^CotG-0859^ spores against *S. aureus* ATCC43300 was found to be equivalent to 19.83 μg of Lys0859 (Supplementary Figure S4d).

### 3.5 *In vivo* pathogen challenge test

**Figure 4:**
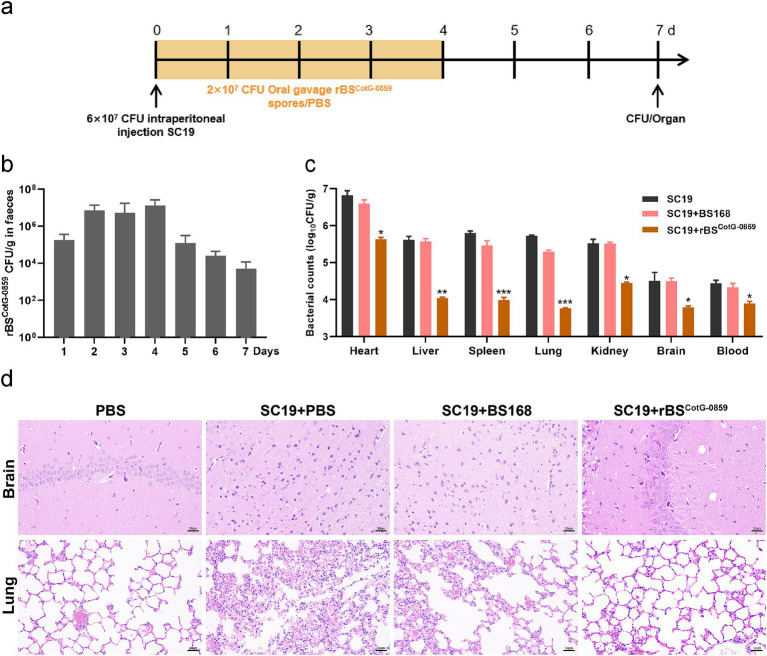
Oral rBS^CotG-0859^ spores alleviated the infection in mice with *S. suis* SC19. **(a)** Experimental design for treatment in this study. Orally administrated with either PBS, BS168, or rBS^CotG-0859^ spores by gavage at days 1, 2, 3, and 4 after *S. suis* SC19 infection (6 × 10^7^ CFU/mouse), respectively. All mice were euthanized at day 7 after *S. suis* SC19 infection. **(b)** Bacterial count of rBS^CotG-0859^ in mouse feces. Fecal samples were collected per day after rBS^CotG-0859^ treatment and resuspended in sterile PBS (0.1 g of fecal resuspended in 1 mL of sterile PBS). Each sample performed a serial of 10-fold dilutions and spread on selective agar plates (10 μg/mL chloramphenicol) and incubated at 37°C for 12 h before bacterial counting. **(c)** The bacterial loads of *S. suis* SC19 in heart, liver, spleen, lungs, kidneys, brain, and blood. **(d)** H&E-stained brain and lung tissue sections. Scale bar: 50 μm.

To evaluate the antibacterial efficacy of rBS^CotG-0859^ *in vivo*, we challenged mice with the *S. suis* SC19 and treated with rBS^CotG-0859^ spores by oral gavage (Figure 4a). As shown in Figure 4b, at least 10^6^ CFU/g of rBS^CotG-0859^ was detected in feces of mice at day 1–4 after post-infection. Subsequently, the amount of rBS^CotG-0859^ in mouse feces showed a progressively decreasing trend from day 5 to day 7. The treatment of rBS^CotG-0859^ spores reduced the SC19 in the heart, liver, spleen, lungs, kidneys, brain, and blood of mice by 1.14, 1.53, 1.81, 1.96, 0.97, 0.47, and 0.52 logs, respectively (Figure 4c), compared to PBS-treat group. Meanwhile, the treatment of rBS^CotG-0859^ spores decreased the SC19 in the heart, liver, spleen, lungs, kidneys, brain, and blood of mice by 0.93, 1.50, 1.43, 1.53, 1.07, 0.69, and 0.39 logs, respectively (Figure 4c), compared with the BS168 treatment group. Histology revealed that the rBS^CotG-0859^ spores significantly relieved brain and lung inflammation and pathological damages such as inflammatory cell infiltrates, alveolar thickening, alveoli interstitial congestion, and edema in the brain and lung of infected mice (Figure 4d).

**Figure 5:**
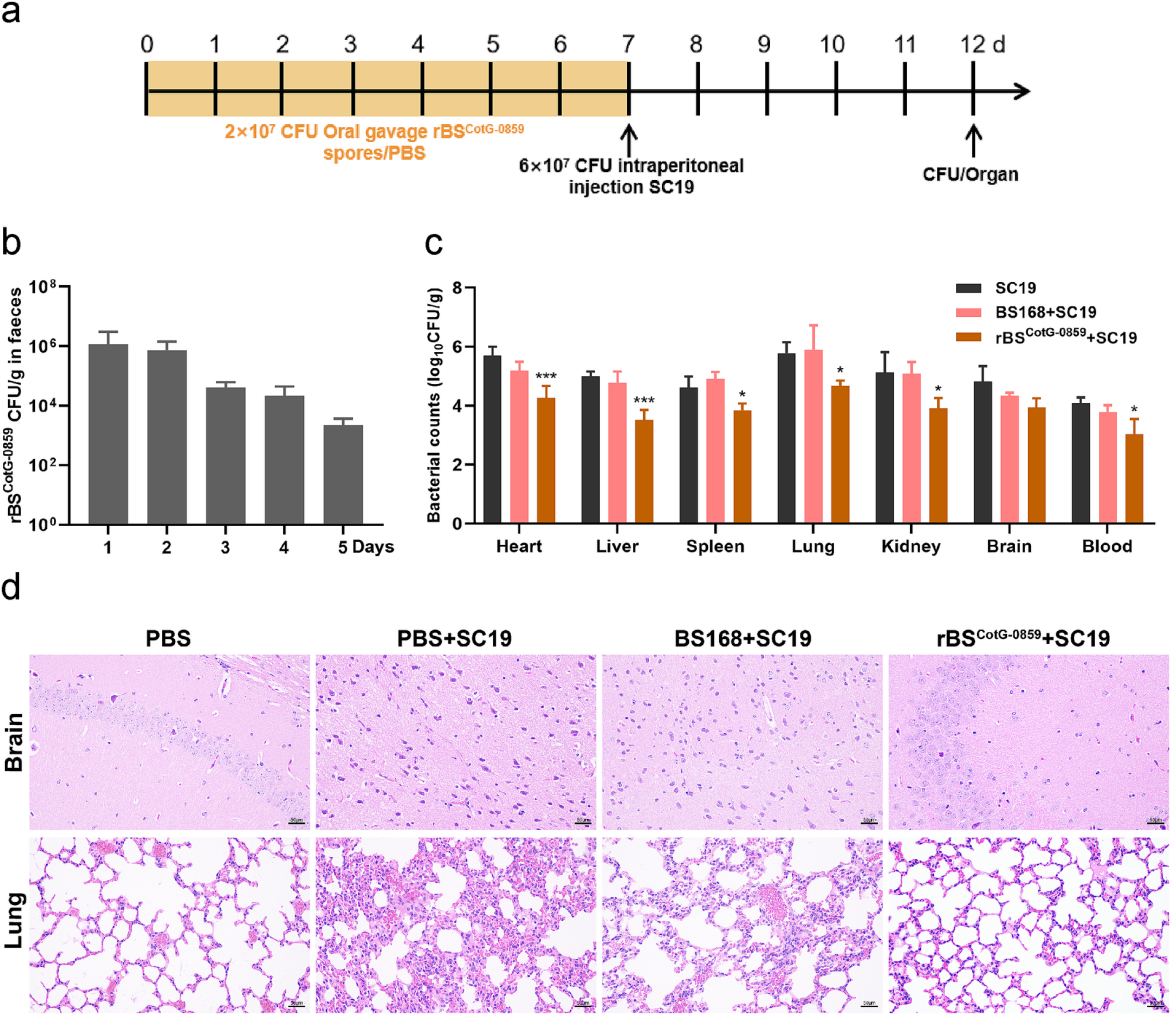
Prophylactic rBS^CotG-0859^ spores attenuated the infection in mice with *S. suis* SC19. **(a)** Experimental design for treatment in this study. At days 1 to 7, each mouse in the PBS + SC19 group, BS168 + SC19 group and rBS^CotG-0859^ + SC19 group were received 200 μL (2 × 10^7^ CFU/mouse) of sterile PBS, BS168 and rBS^CotG-0859^ spores by gavage, respectively. Then, mice in PBS + SC19 group, BS168 + SC19 group and rBS^CotG-0859^ + SC19 group were intraperitoneal injected with 200 μL (6 × 10^7^ CFU/mouse) of *S. suis* SC19 on day 7. On day 12, all mice were euthanized. **(b)** Bacterial count of rBS^CotG-0859^ in mouse feces. **(c)** The bacterial loads of *S. suis* SC19 in heart, liver, spleen, lungs, kidneys, brain, and blood. **(d)** H&E-stained brain and lung tissue sections. Scale bar: 50 μm.

Based on the enhanced efficacy of rBS^CotG-0859^ spores in the treatment of SC19 infection, the potential of disease prevention was further explored through pre-treatment (Figure 5a). As shown in Figure 5b, the number of rBS^CotG-0859^ in mouse feces showed a gradually decreasing trend from day 8 to day 12 of the experiment, which is similar to the results of the therapeutic experiment. The prophylactic treatment of rBS^CotG-0859^ spores reduced the SC19 in the heart, liver, spleen, lungs, kidneys, brain, and blood of mice by 1.42, 1.48, 0.77, 1.09, 1.21, 0.87, and 1.05 logs respectively, compared to the group pretreated with PBS (Figure 5c). Similarly, the prophylactic treatment of rBS^CotG-0859^ spores declined the SC19 in the heart, liver, spleen, lungs, kidneys, brain, and blood of mice by 0.92, 1.25, 1.05, 1.21, 1.16, 0.38, and 0.75 logs respectively, compared with the BS168 pretreated group (Figure 5c). The results of histological analysis further that oral administration of rBS^CotG-0859^ spores significantly reduced the severity of brain and lung injury (Figure 5d), consistent with the overall results of the therapeutic trial.

## 4 Discussion

*S. suis* is an important zoonotic pathogen that cause systemic infection in pigs as well as humans (Xia et al., 2019), which not only lead huge economic losses in the pig industry, but also pose a threat to the public health (Pei et al., 2020). Although bacterial infections are commonly treated with antibiotics, the overuse of antibiotics contribute to the development of antibiotic resistance in recent years (Laxminarayan et al., 2013). Therefore, a promising alternative to antibiotics is particularly important.

An increasing number of studies demonstrated that endolysins encoded by bacteriophage is a potentially attractive method of treating bacterial infections. For example, Li et al. reported that prophage lysin Lys0859 could significantly reduce the bacterial load of *Streptococcus agalactiae* in mouse mammary glands, and also significantly improve the survival of *S. suis* mice with systemic infection (Li et al., 2023). Lood et al. also reported the novel phage lysin PlyF307 could effectively kill multidrug-resistant *Acinetobacter baumannii* in mice, thereby rescuing mice from lethal bacteremia (Lood et al., 2015). In addition, some chimeric lysins exhibited a stronger bactericidal activity (Díez-Martínez et al., 2015; Briers et al., 2014; Duan et al., 2023). However, by its very nature, lysin is formed from polypeptides or proteins by folding, which may result in the rapid degradation and inactivation after introduced into the gastrointestinal tract. It is well known that bacillus endospores are highly resistant to many physical and chemical assaults and are able to persist in complex environment. In this study, *B. subtilis* endospores were used as a microparticle platform, introducing prophage lysin Lys0859 on the surface of the spores. It was discovered that the resistance of tethered lysin Lys0859 against environmental assaults outperformed that of the free lysin Lys0859. Similarly, the haloalkane dehalogenase DhaA displayed on *B. subtilis* spores exhibited enhanced stress resistance and activity compared to free DhaA in harsh chemical environments (Wang F. et al., 2019). Particularly, to our knowledge, Lys0859 was the first phage lysin to exhibit highly effective bactericidal activity by displaying on the surface of *B. subtilis* endospores. Furthermore, we have found that the growth rate and spore generation rate of recombinant bacteria are consistent with those of wild strains.

Although *B. subtilis* spores significantly enhanced the extreme resistance and antibacterial activity of Lys0859, stably passaged of recombinant *B. subtilis* are also crucial for clinical application from a genetic perspective. The efficacy of recombinant *B. subtilis* in exerting bactericidal effects is primarily contingent upon the presence of exogenous fusion protein on its spore surface. Nevertheless, the inadvertent loss of the target gene fragment during successive passaging may significantly compromise the antibacterial potency of the recombinant bacteria, thereby impeding its clinical utility. Hence, the stable passaging and expression of exogenous gene fragments is particularly important. The findings of this investigation demonstrate that the fusion gene remained intact and continued to demonstrate robust antibacterial or bactericidal properties even after 10 consecutive passages of the recombinant bacteria.

It has been reported that Lys0859 exhibits excellent antibacterial activity against multiple serotypes of *S. suis*, especially *S. suis* SC19 (serotype 2). In this study, we obtained consistent experimental results that support the results previously reported by Li et al. (2023). Because we have found that the recombinant spores displaying Lys0859 on the surface can effectively kill multiple *streptococci*, including *S. suis*, *S. agalactiae*, and *S. dysgalactiae*. More specifically, based on the agar well diffusion assay, we found that the antibacterial potency of rBS^CotG-0859^ spores with 1 × 10^6^ CFU against *S. suis* SC19, *S. suis* SS3, *S. agalactiae* ATCC13813, and *S. dysgalactiae* SD002 were equivalent to 39.11, 44.52, 25.85, and 46.71 μg of Lys0859, respectively. The surface display of *B. subtilis* spores not only compensates for the deficiencies of Lys0859 stress resistance, but also maintains its original bactericidal activity, which providing a feasible alternative for application of bacteriophage lyases in the clinical. Moreover, the application of rBS^CotG-0859^ may greatly reduce the widespread use of antibiotics in livestock and poultry breeding industry. More importantly, Lys0859 is a biodegradable protein that will not remain and accumulate in livestock and poultry.

In order to investigate the antibacterial activity of recombinant bacteria against *S. suis* SC19 *in vivo*, we established a mouse model of *S. suis* SC19 infection. The results showed that 4 days after treatment with rBS^CotG-0859^ spores (2 × 10^7^ CFU), the bacterial loads had decreased 0.47 to 1.96 logs (*p* < 0.05) in all organs and blood tested for *S. suis*. Similarly, *streptococcal* prophage Ply30 lysin reduced the load of *S. suis* in all organs and blood of mice by 3 to 5 logs (*p* < 0.01) (Tang et al., 2015). Besides, Li et al. found that the intraperitoneal injection of 100 μg/mouse of Lys0859 at 1 h post-infection decreased the load of *S. suis* SC19 in all organs and blood of mice by 0.57–1.39 logs (Li et al., 2023). Next, we evaluated the prophylactic efficacy of rBS^CotG-0859^ on *S. suis* SC19 infection through pretreatment. The results revealed rBS^CotG-0859^ prevention trials obtained similar results as the treatment, such as significantly reduced the load of *S. suis* in various organs. In summary, this study revealed that the efficacy of lysins anchored to the surface of rBS^CotG-0859^ spores outperform that of the free enzymes.

Although this study has demonstrated that rBS^CotG-0859^ spores can significantly reduce the bacterial load in infected tissues and improve pathological damage, immune efficacy evaluation remains a crucial missing piece in the puzzle of our research. However, the dynamic changes in the host immune response are still a key dimension for evaluating its therapeutic efficacy. Previous experiments have shown that Lys0859 can reduce the levels of TNF-α and IL-6 in mice after mastitis infection (Li et al., 2023). *B. subtilis* 168-CLE can effectively increase the levels of IgA and IgG in mice, indicating that the COE displayed on the spore surface has the ability to stimulate mucosal immunity and the production and secretion of more antigen-specific antibodies by B cells (Tian et al., 2024). In the subsequent experimental plan, it is necessary to measure cytokine and antibody levels, analyze immune cell subsets, and study the distribution and activation of immune cells in infected tissues. By integrating these immune-related data with our existing results on tissue bacterial load and pathological changes, we can draw more comprehensive and accurate conclusions about the effectiveness and mechanism of action of our treatment.

## 5 Conclusion

In conclusion, in this study, we successfully displayed prophage lysin Lys0859 on the surface of *B. subtilis* spores using CotG as an anchor protein, and demonstrated excellent bactericidal activity against Streptococci *in vitro*. On the other hand, our experimental results strongly demonstrated that the surface display of *B. subtilis* spores not only significantly enhances the stress resistance of Lys0859, but also maintains the bactericidal activity of Lys0859. Most importantly, the surface display of *B. subtilis* spores not only reduces complex and time-consuming preparation and purification steps, but also reduces the costs, this lays the foundation for the clinical application of prophage lysin.

## Data Availability

The raw data supporting the conclusions of this article will be made available by the authors, without undue reservation.
