# Supplementary material for: Surface display of Lys0859, a Streptococcus suis prophage lysin, on Bacillus subtilis spores and its antibacterial activity against Streptococcus suis
Source: Front Microbiol. 2025 Mar 24;16:1519935. doi: 10.3389/fmicb.2025.1519935 (PMC11973311; doi:10.3389/fmicb.2025.1519935)
Supplement: Supplementary file 1 [file Data_Sheet_1.doc]

**Supplementary Data**

**Supplementary Figures**


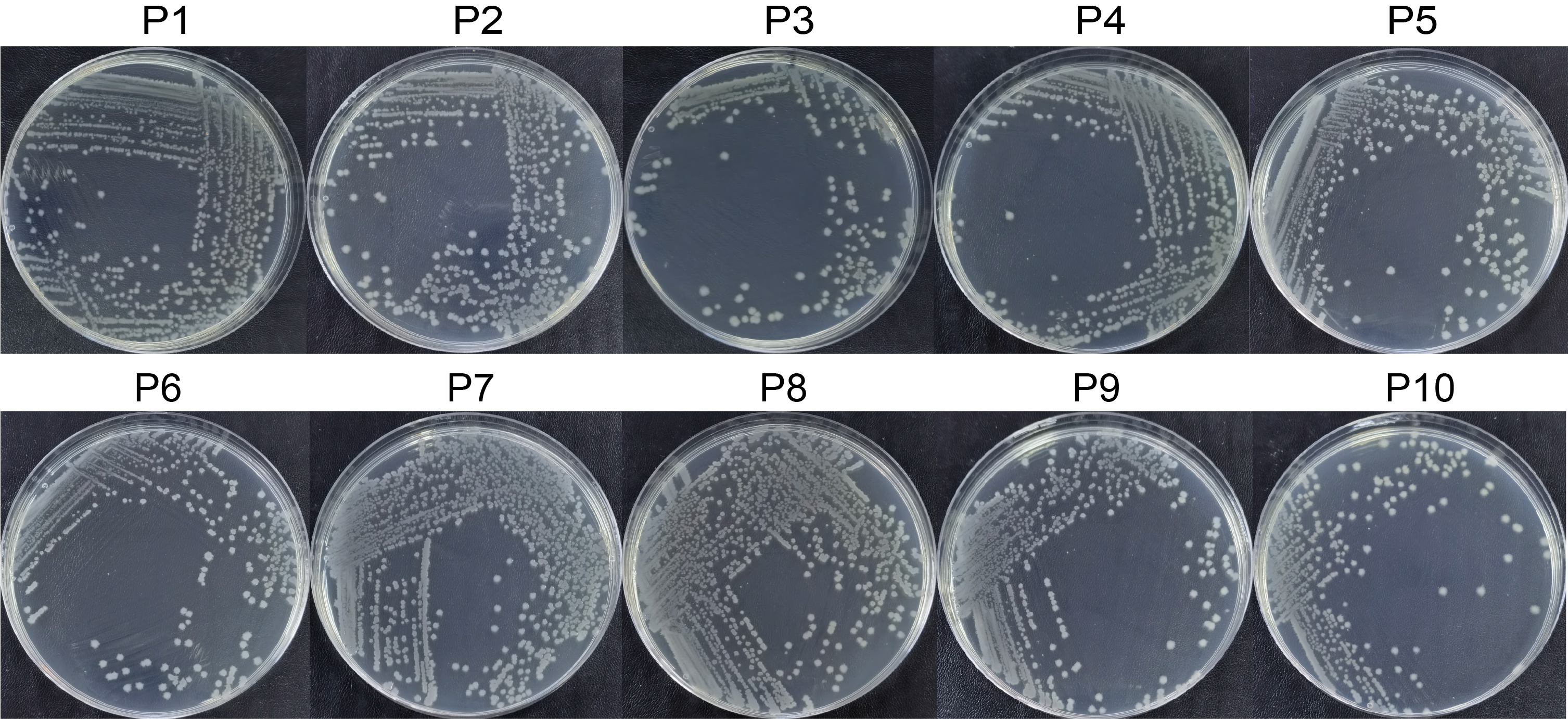


**Fig. S1 Genetic stability of rBS^CotG-0859^.** The morphology of the rBS^CotG-0859^ from the 1st to the 10th generation on LB agar plates.


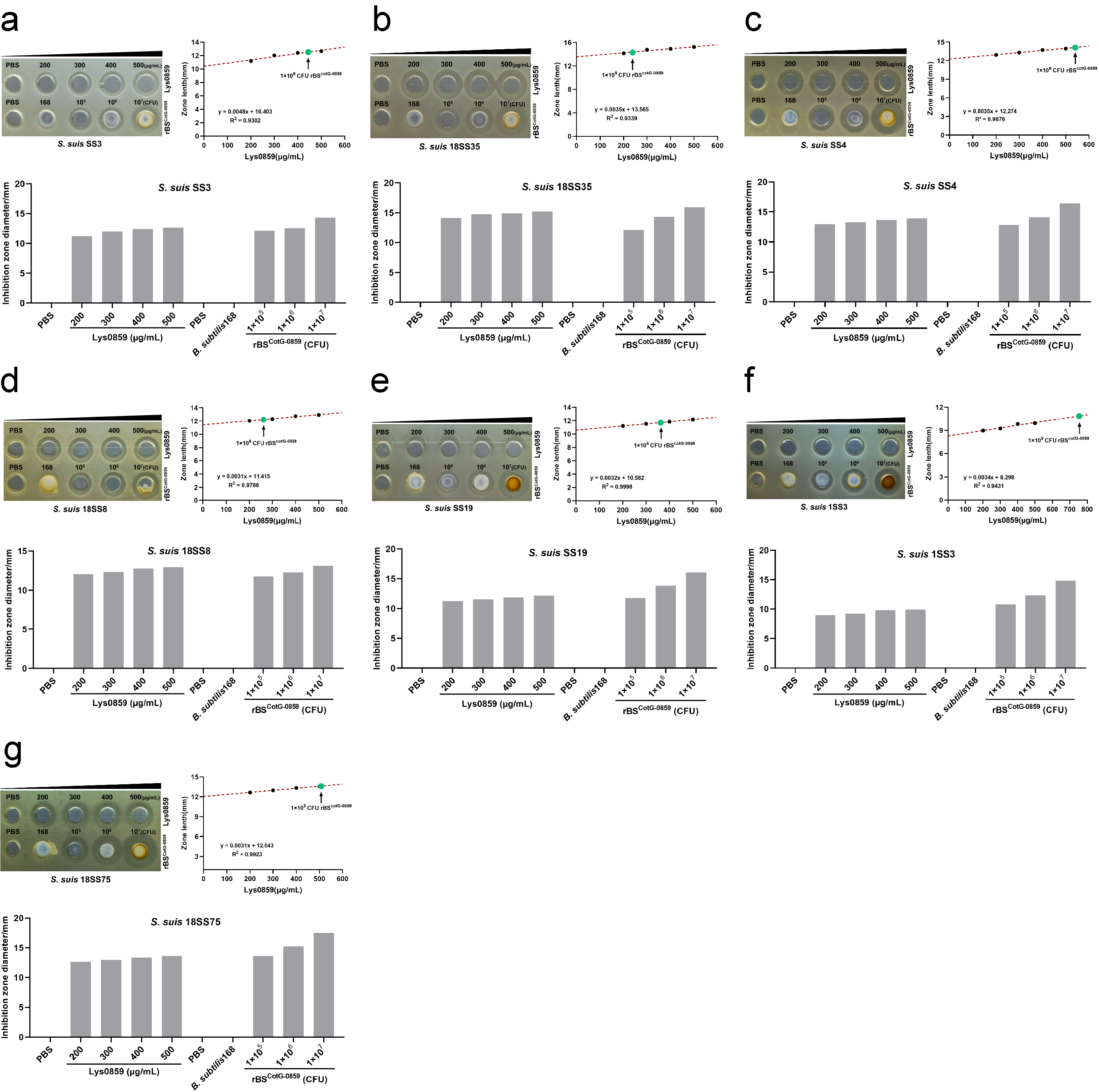


**Fig.S2 Bactericidal activity of the rBS^CotG-0859^**. The antibacterial efficacy of recombinant Lys0859 produced by rBS^CotG-0859^ spores against (**a**) *S. suis* SS3, (**b**) *S. suis* 18SS35, (**c**) *S. suis* SS4, (**d**) *S. suis* 18SS8, (**e**) *S. suis* SS19, (**f**) *S. suis* 1SS3, and (**g**) *S. suis* 18SS75.


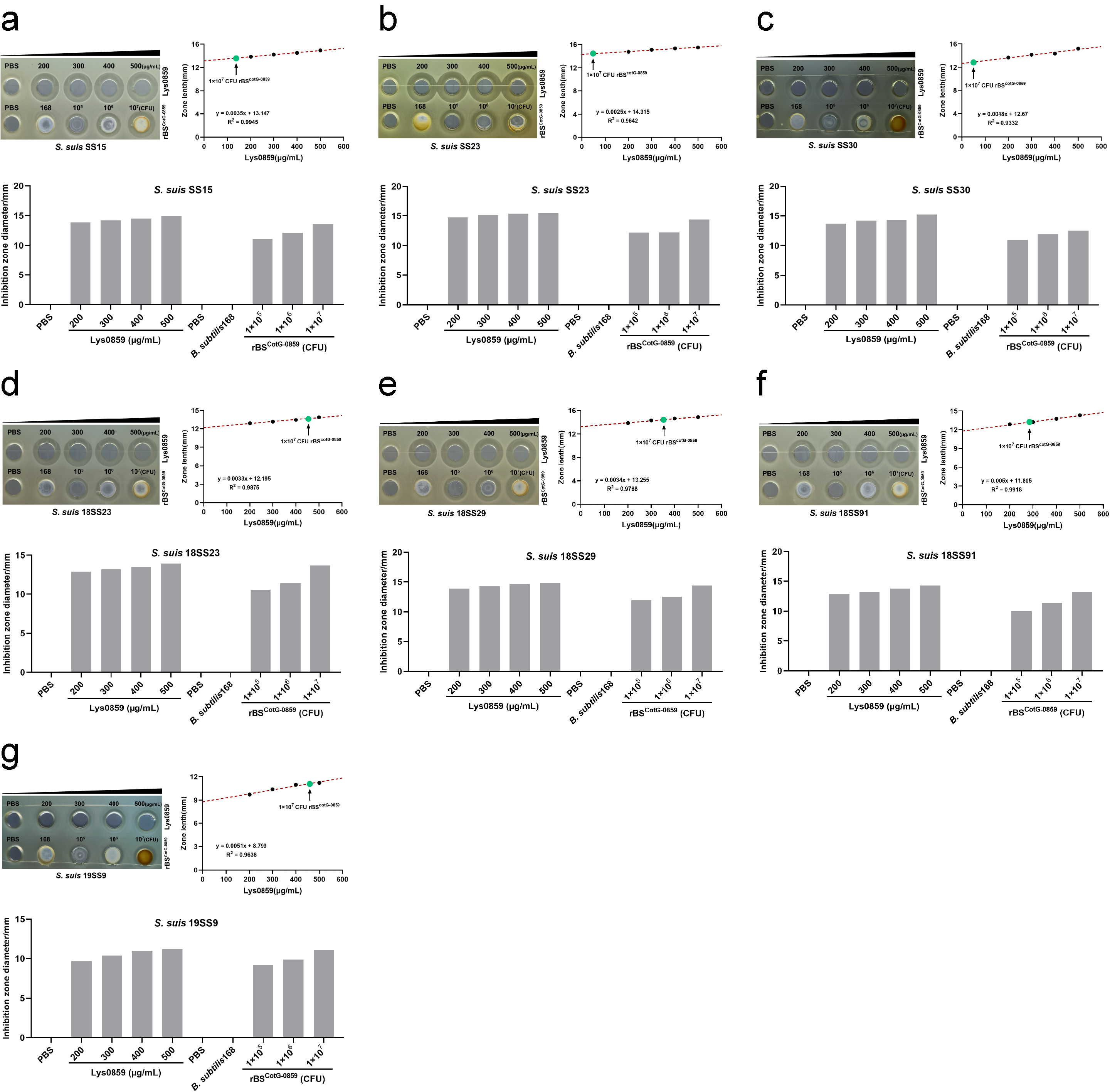


**Fig. S3** **Bactericidal activity of the rBS^CotG-0859^**. The antibacterial efficacy of recombinant Lys0859 produced by rBS^CotG-0859^ spores against (**a**) *S. suis* SS15, (**b**) *S. suis* SS23, (**c**) *S. suis* SS30, (**d**) *S. suis* 18SS23, (**e**) *S. suis* 18SS29, (**f**) *S. suis* 18SS91, and (**g**) *S. suis* 19SS9.


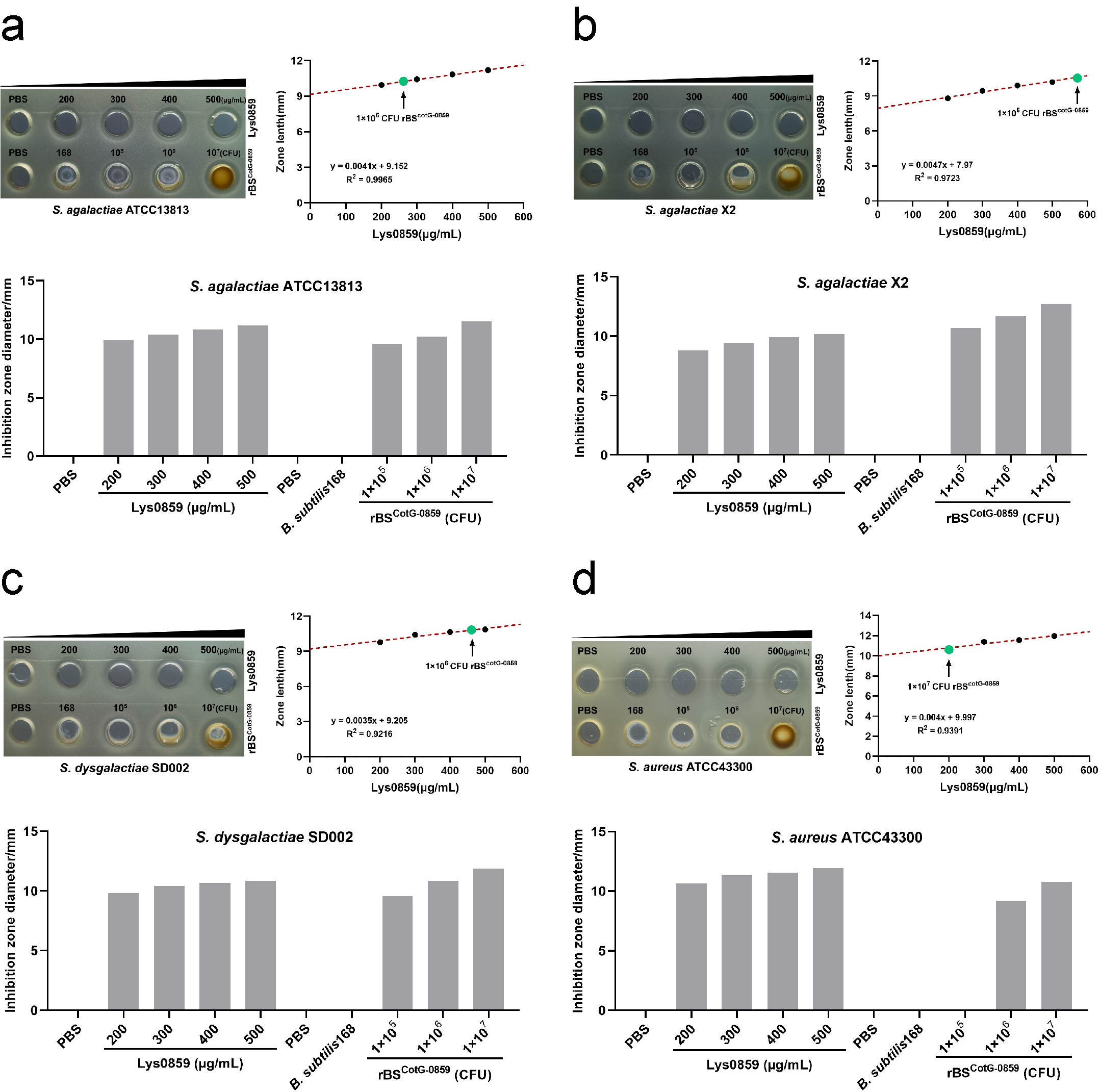


**Fig. S4 Bactericidal activity of the rBS^CotG-0859^**. The antibacterial efficacy of recombinant Lys0859 produced by rBS^CotG-0859^ spores against (**a**) *S. agalactiae* ATCC13813, (**b**) *S. agalactiae* X2, (**c**) *S. dysgalactiae* SD002, and (**d**) *S. aureus* ATCC43300.

**Supplementary Tables**

**Table S1. The strains used in this study**

| **Species** | **Strains** | **Source** |
| --- | --- | --- |
| *S. suis^a^* | Serotype 2, SC19 | Swine |
|  | Serotype 2, SS0859 | Swine |
|  | Serotype 2, 18SS23 | Swine |
|  | Serotype 3, 18SS29 | Swine |
|  | Serotype 4, 18SS75 | Swine |
|  | Serotype 5, 18SS91 | Swine |
|  | Serotype 7, SS3 | Swine |
|  | Serotype 13, 19SS9 | Swine |
|  | Serotype 16, SS23 | Swine |
|  | Serotype 18, SS4 | Swine |
|  | Serotype 21, 18SS35 | Swine |
|  | Serotype 24, SS19 | Swine |
|  | SS15 | Swine |
|  | SS30 | Swine |
|  | 1SS3 | Swine |
|  | 18SS8 | Swine |
| *S. agalactiae^a^* | ATCC13813 | Bovine |
|  | X2 | Bovine |
| *S. dysgalactiae^a^* | SD002 | Bovine |
| *S. aureus^b^* | ATCC43300 |  |
| *B. subtilis* | 168 |  |
|  | CotG-0859 |  |
| *E. coli^b^* | DH5α |  |
|  | BL21 |  |

^a^These strains sources were isolated from bovine and swine in our lab.

^b^The strains were purchased from ATCC and commercial company

**Table S2. Plasmids and Primers used in the study**

| **Plasmids and Primers** | **Squences (5'-3')** |
| --- | --- |
| **Plasmids** |  |
| pDG364 | *E. coli*-*B. subtilis* shuttle vector |
| pDG364CotG-0859 | pDG364 derivative carrying the fusion CotG-0859 gene |
| pCold II | Cold shock expression vector pCold TF |
| pCold-0859 | pCold derivative carrying the 0859 gene |
| pCold-CotG-0859 | pCold derivative carrying the fusion CotG-0859 gene |
| **Primers** |  |
| CotG-F | GGATCCATGTTGGGCCACTATTCCCATTCTGACATCGAAG |
| CotG-R | TCGATCCAGACGAGCCTCCTTTGTATTTCTTTTTGACTACCCAGCA |
| 0859-F | AAGGAGGCTCGTCTGGATCGATGACAACAGTATTTGAAGTAGTCAA |
| 0859-F1 | GGATCCATGACAACAGTATTTGAAGTAGTCAAT |
| 0859-R | GAATTCTTAGTGGTGGTGGTGGTGGTGTTTGAAAATACCATAAGGC |
| 0859-R1 | AAGCTTTTAGTGGTGGTGGTGGTGGTGTTTGAAAATACCATAAGGC |
| AmyE-F | GGGATTTTTGACTCCGAAGTAAGTC |
| AmyE-R | GGTAAGTCCCGTCTAGCCTTGC |
| G8-F | TTACGCGAAATACGGGCAGACAT |
| G8-R | TTTTTAAAGGATTTGAGCGTAGCG |
| 168-F | CATTGATTTGTATTCACTCTGCCAAGTTG |
| 168-R | CATCAATGACCACAAGCTCATCTGTGAT |
